# Exploring barriers and facilitators for mental health professionals delivering behavioural activation to young people with depression: qualitative study using the Theoretical Domains Framework

**DOI:** 10.1192/bjo.2022.7

**Published:** 2022-02-04

**Authors:** Kate Whittenbury, Leopold Kroll, Bernadka Dubicka, Eleanor R. Bull

**Affiliations:** Faculty of Health and Education, Manchester Metropolitan University, UK; Young People's Mental Health Research Unit, Pennine Care NHS Foundation Trust Headquarters, UK; Young People's Mental Health Research Unit, Pennine Care NHS Foundation Trust Headquarters, UK; and Faculty of Biology, Medicine and Health, University of Manchester, UK; Faculty of Health and Education, Manchester Metropolitan University, UK; and Department of Anaesthesia, Manchester University NHS Foundation Trust, UK

**Keywords:** Behavioural activation, children and young people, theoretical domains framework, implementation, depression

## Abstract

**Background:**

Depression prevalence among young people is increasing, with growing pressures on specialist mental health services. Manualised behavioural activation therapy may be effective for young people, and can be delivered by a range of mental health professionals (MHPs). This study explored clinician perspectives of barriers and facilitators to implementing behavioural activation with young people in routine practice.

**Aims:**

We conducted a qualitative study with individual semi-structured interviews with MHPs, as part of a wider feasibility study.

**Method:**

Participants were mental health professionals (therapists and supervisors) from two UK NHS sites delivering manualised behavioural activation for young people. Data were analysed with an inductive followed by deductive approach, applying the Theoretical Domains Framework (TDF) to understand key influences on practice change. Identified domains were mapped onto possible behaviour change techniques (BCTs) to support implementation, using the Theory and Techniques Tool (TTT).

**Results:**

Nine MHPs were interviewed. Thirteen of fourteen TDF domains were relevant, including perceived professional identity, beliefs about own capabilities and perceived positive or negative consequences of using manualised behavioural activation, social influences, memory and attention, and environmental resources. Fourteen theory-linked BCTs were identified as possible strategies to help clinicians overcome barriers (e.g. integrating behavioural practice/rehearsal, prompts and persuasive communications within training, and supervision).

**Conclusions:**

Behavioural science approaches (TDF, TTT) helped conceptualise key barriers and facilitators for MHPs delivering manualised behavioural activation with young people. Interventions using BCTs to address identified barriers could help the implementation of new therapies into routine practice, working to bridge the research–practice gap in clinical psychology.

Prevalence rates of depression among young people are rising worldwide.^1^ Services remain under-resourced and understaffed, with experts arguing that we are experiencing a children's mental health crisis.^2^ Currently, in the UK, National Institute for Health and Care Excellence clinical guidelines^3^ for young people with moderate-to-severe depression recommend 12 weeks of cognitive–behavioural therapy (CBT) within a specialist Child and Adolescent Mental Health Service (CAMHS), typically including both cognitive and behavioural treatment approaches aiming to help young people think and act differently to break negative thinking and behaviour patterns thought to maintain depression. Behavioural activation is a more behaviourally oriented psychological treatment focusing on helping a person do more activities that are valued (inherently rewarding) in a structured and achievable way, to improve mood. Behavioural activation has an established evidence base as an alternative to CBT in studies with adults,^4^ and is proving promising for young people.^5^ Importantly, manualised behavioural activation can be delivered by mental health professionals (MHPs) without in-depth specialist training, and so may be less costly than traditional CBT.^6^ Although available in some CAMHS, in the UK as in other countries, behavioural activation is not being implemented consistently or in a structured way.^7^ It is important to study implementation factors, such as professionals’ perceptions of delivering behavioural activation particularly in the current global mental health context.^8^ To implement new therapies and evidence into routine practice, MHPs often need to make substantial changes to their practice behaviours at work.^9^ However, the research–practice gap in healthcare (including mental healthcare) is well known.^10^ It is estimated that 30-40% of patients do not receive evidence-based practice, with as much as 25% of healthcare given being needless or even damaging.^11^ Applying behavioural science to identify barriers and facilitators can provide clear explanations of mechanisms of change, and can assist researchers in understanding why health professionals do or do not change their behaviour and, consequently, why interventions are or are not implemented effectively.^12^ Several tools have been recently developed by groups of psychological researchers aiming to help others understand and facilitate implementation of new therapies and evidence in practice. One of these tools is the Theoretical Domains Framework (TDF), a synthesis of 33 behavioural theories of behaviour change forming 14 domains that create a framework that researchers can use as a theoretical basis to understand cognitive, social, emotional and environmental influences on healthcare professionals’ behaviour.^13^ The TDF has been used extensively in implementation research investigating facilitators and barriers of implementing interventions within various healthcare settings.^9,14^ National Institute for Health and Care Excellence guidelines suggest research should investigate which behaviour change techniques (BCTs) are most likely to stimulate and support behaviour change, resulting in successful implementation of new interventions into routine practice.^15^ However, this process has not been widely used in clinical psychology, and has never been used to explore MHP implementation of behavioural activation for young people. Another is a taxonomy of 93 BCTs, defined as ‘active components of an intervention designed to change a behaviour’.^16^ BCTs can be used as practical, theory-linked strategies to address identified barriers to behaviour change in a defined context, leading to better implementation practices and thus patient outcomes.^17^ The most recent behavioural science development, the Theory and Techniques Tool (TTT),^18^ was developed to assist with this linkage process.

This study applied the TDF and TTT to explore MHPs’ perceived barriers and facilitators of delivering manualised behavioural activation in two National Health Service (NHS) sites, as part of a wider feasibility study. This study aimed to understand implementation barriers and facilitators perceived by MHPs implementing manualised behavioural activation with young people in CAMHS settings, using the TDF; and map relevant TDF domains to BCTs by using the TTT to determine relevant intervention components that could support MHPs’ implementation of manualised behavioural activation in future.

## Method

The Consolidated Criteria for Reporting Qualitative Research (COREQ) was used to guide this qualitative investigation.^19^ This study explored perspectives of MHPs delivering behavioural activation as part of a mixed-methods feasibility study. The wider feasibility study details, fidelity measures and participant outcomes are reported in full elsewhere.^20,21^

In brief, seven MHPs delivered eight sessions of manualised behavioural activation to 33 young people aged 12–17 years, over 10 months (July 2019 to April 2020), in two specialist NHS CAMHS sites within the North-West of England. CAMHS are the UK NHS mental health services for children and young people, delivering evidence-based psychological therapies to help ameliorate affective or behavioural problems. The eight behavioural activation workbooks and programme overall (training, implementation, delivery) were co-produced with young people and clinicians, with the first four focusing on orientation of the behavioural activation model, values and goals, and activity scheduling; and the remainder focusing on the role of avoidance, overcoming barriers, future planning and depression relapse prevention. MHPs were offered a training day followed by half a day of training and MHP discussion days every 3 months, alongside regular clinical supervision.

For this study, using a homogeneous purposive sampling technique, all MHPs participating in the wider study were invited by letter to take part in a semi-structured interview surrounding their experience during the active study timeframe. An open-ended question topic guide was developed^22^ to explore in-depth perspectives of barriers and facilitators to implementing behavioural activation with young people (see Supplementary File 1 available at https://doi.org/10.1192/bjo.2022.7). The researcher was independent from the wider feasibility study team, had previous experience of conducting qualitative research and interviews and was trained in research methods to a Master's degree level. The interviewer established relationships with the participants implementing the new behavioural activation intervention before data collection began, and kept a reflexive journal throughout the research project. Written informed consent was obtained from all participants. Interviews were audio-recorded with a password-protected mobile phone, with the participant's consent. Audio recordings taken within interviews were transcribed onto an encrypted computer within 4 days of the original interview, and the recording was deleted. All identifiable information was anonymised in transcripts to secure participants’ privacy. The authors assert that all procedures contributing to this work comply with the ethical standards of the relevant national and institutional committees on human experimentation and with the Helsinki Declaration of 1975, as revised in 2008. All procedures involving human patients were approved by the Health Research Authority's Integrated Research Application System (project 257613).

### Data analysis

An experimental approach to thematic analysis was used to analyse the interview transcripts. The TDF was used as a structure to analyse and interpret data.^23^ Two coders independently followed^24^ Six phases of thematic analysis^25^ alongside those within a guide to using the TDF for implementation studies.^26^ Familiarisation with the data-set began through transcription and by re-reading transcripts. Then, each transcript was inductively coded and code names were generated to identify points of interest in the data relevant to the research question.^24^ Code names were then matched to domains of the TDF, following previous implementation studies using the TDF (e.g. Alexander et al^27^).

Additionally, to identify BCTs to support implementation, key domains were mapped onto BCTs by the novel TTT.^18^ This visually represents the strength of evidence for 74 BCTs to help improve each TDF domain (labelled mechanisms of action in the TTT). The strength of these links (between BCTs and mechanisms of action) was established by a literature review of 277 behaviour change studies^28^ and an expert study of consensus.^29^ In the present study, 39 BCTs were identified and then checked with the APEASE (affordability, practicality, effectiveness, acceptability, side-effects, equity) criteria,^30^ a tool used to access the feasibility of BCTs and interventions in real-life contexts.^31^ Research studies usually concentrate on the effectiveness of interventions. The APEASE criteria are used to assess if such interventions are feasible in real clinical practice situations, and can be used to modify, prioritise or omit parts of the intervention.^30^

The lead study author evaluated each potential BCT against each APEASE criterion, with strong reference to the practical suggestions gathered from clinicians in the interviews, and checked them with the study leads, a team of experienced academic–practitioner psychologists and psychiatrists.

## Results

### Interviews and participant characteristics

Nine face-to-face interviews were conducted, lasting 20–50 mins at each participant's place of work. All participants were NHS MHPs. Seven participants were ‘implementation staff’ (i.e. MHPs delivering the therapy), including three assistant psychologists and four psychological well-being practitioners. The two other participants were supervisors of the implementation staff: a CBT therapist and a clinical psychologist. Participants were based at one of two NHS CAMHS in the North of England (termed site A and site B for anonymity). Participants were aged between 21 and 45 years, eight out of nine were female, and all participants had English as their first language. Implementation staff varied in experience levels with behavioural activation similarly to variation seen in real-life CAMHS teams. Two professionals at two different sites had used a different behavioural activation manual before. The five other professionals had no prior experience of behavioural activation delivery.

Full details of patients included are published in the outcome paper.^20^ In brief, 33 young people aged 12–17 years were involved; 11 were male (33%) and 22 were female (67%); and patients were of mixed (White and Asian) (*n* = 2), Asian (*n* = 2), Black (African) (*n* = 1) or White British (*n* = 28) ethnicity.

### Barriers and facilitators to implementation

**Fig. 1:**
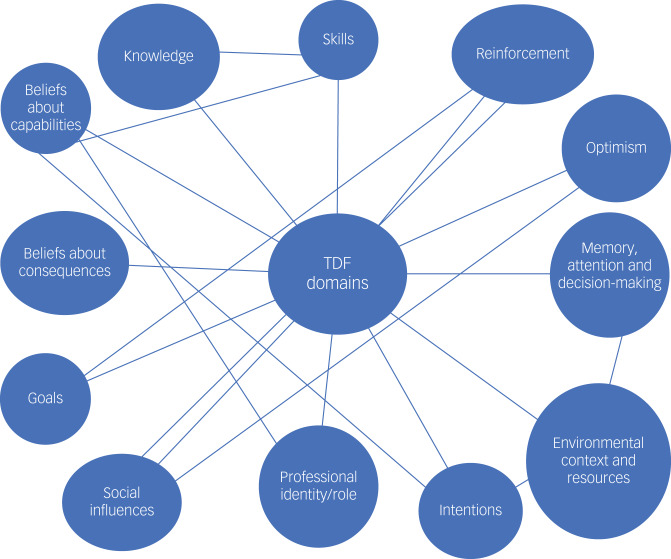
Thematic map demonstrating interactions between identified TDF domains. TDF, Theoretical Domains Framework.

Interviews captured a diverse range of perceived barriers and facilitators to implementation of behavioural activation with young people. Initial codes mapped onto 13 out of 14 TDF domains identified as relevant, including beliefs about capabilities; professional identity and role; social influences; beliefs about consequences; memory, attention and decision-making; environmental resources and context; intentions; knowledge; skills; reinforcement; optimism and emotions (see Table 1). As explored below, domains interacted with one another (see Fig. 1). Beliefs about capabilities and professional roles/identity were dominant domains in the data and often interlinked, suggesting that how an MHP viewed themselves and their capabilities was interconnected, and influenced the likelihood of a clinician using the new intervention. Another central domain identified throughout the interviews was beliefs about consequences. Intervention facilitators’ use of the new programme was influenced by their perceptions of how effective the intervention would be and whether the manualised therapy was ‘enough’ as a standalone intervention for a particular young person. Also, social influences were found to influence the uptake and implementation of the new intervention by MHPs. The perceived social influences differed between the two NHS centres, which affected the implementation of the behavioural activation intervention differently. Overall, participants perceived the new intervention positively, but implementation was subject to a number of influences. Key domains of interest are summarised below.

**Table 1:** Allocation of codes onto domains of the Theoretical Domains Framework

TDF domain	Barrier or facilitator	Codes	Example quotes
Knowledge	Facilitator	Rationale for using behavioural activation	‘Erm, to be honest I have not had a lot of experience in using behavioural activation so it was quite interesting in the training, I was a bit tentative before, because of not having the prior knowledge.’ (Clinician 104) ‘I think because I get the whole idea around behavioural activation that's helped me a bit I think if I was coming to it completely fresh, I would be a bit disorientated because of the bubbles and stuff all over the place.’ (Clinician 108)
		Previous knowledge of behavioural activation	
		Behavioural activation simplistic model	
		Previous evidence of behavioural activation	
Skills	Facilitator or barrier	Previous experience of using behavioural activation	‘I've gone through IAPT course, part of my training was using behavioural activation for low mood and depression anyway, which was something that I have been doing for 2 years’ (Clinician 103) ‘I think it depends on the skill set of the person, they are going to need. If I hadn't used behavioural activation before, I think counselling skills help and I think pastoral people using it would need that input because it definitely helps.’ (Clinician 105)
		No previous therapeutic experience	
		Using the manual flexibly	
		Gaining new skills	
		Hung up on techniques	
Professional role and identity	Barrier or facilitator	New professional	‘I am a relatively new clinician, so I was quite apprehensive. I've done an undergraduate degree in psychology and you learn the basics of therapy and a bit of CBT, but I'd never actually done it in practice.’ (Clinician 102) ‘I guess if you are training or just starting out it is really good but if you are well-established in your own therapeutic work following someone else's can be quite difficult.’ (Clinician 108)
		Inexperienced professional	
		Specialised clinician	
		Shaky professional confidence	
		Set in ways	
		Too specialised	
Beliefs about consequences	Barrier or facilitator	Behavioural activation can be effective in treating low mood	‘Yes, so that is a barrier I would say. It's because they are busy and I think because even though we say, “yes, we accept”, comorbidity, there is always feeling that it may needs more or it needs a case manager that can manage it and do the work, rather than getting a case manager and then a second person to do the therapeutic work. I mean also the capacity of the new practitioners we have got.’ (Clinician 107)
		Widen access to therapies	
		Could be used by clinicians	
		Decrease waiting list	
		May not be enough for some clients	
		May be patronising	
		Too simplistic	
		May not work with comorbidities	
		Children and young people need to understand rationale of behavioural activation	
		Age appropriate	
		Older children and young people will not like it	
		Behavioural activation is very repetitive	
Optimism	Facilitator	Positive before starting	‘With staff, I think because the majority of staff here had used behavioural activation before they were already like we are doing this anyway so it might as well go towards the research erm, I think they use a different booklet so there was a feel of let's try something new and I think the clinicians that had been using the other manual for a while were happy for a change and there are a couple of new clinicians like me who were quite nervous but also excited to actually do something with the young people.’ (Clinician 102) ‘I thought it was a really good idea. It's not the first behavioural activation manual I have heard about because I work some of the time on the IAPT for the children and young person training and I knew about a pre-existing behavioural activation manual which was actually being used in this service, so this was another one being brought it, and I was curious but excited, yeah.’ (Clinician 109)
		Excited to try something new	
		Effectiveness of behavioural activation	
		Others like behavioural activation	
Beliefs about capabilities	Barrier or facilitator	Low in confidence	‘I felt it was clunky at first because I wasn't familiar with the material but the more you use it, the more you get to know what is coming. Erm, so I think the more I use it I will feel more comfortable and go a bit off grid with is, so you can bring in your own stuff as well and flip things round and be a bit more confident in doing that.’ (Clinician 105) ‘Erm, because of my training and my particular role, I've gone through IAPT course, part of my training was using behavioural activation for low mood and depression anyway, which was something that I have been doing for 2 years with a slightly altered model.’ (Clinician 103)
		Practice will make me better	
		Need to do everything perfectly	
		Saying the wrong thing	
		Things going wrong	
		In the deep end	
		High in confidence	
		Dip in and out	
		Stuck in ways	
		Invalidates old ways	
		Getting stuck	
Reinforcement	Facilitator	Advancement in career	‘I think because their interest is getting on to a clinical psychology doctorate, they are quite happy to a clinical intervention around that and they are quite open and have that mind set.’ (Clinician 103) ‘I think some services you have to be really really well trained to do it but then there obviously isn't that many clinicians, so I think it's better for sort of new clinicians to be able to do something because then more people are getting seen.’ (Clinician 102)
Intentions	Barrier	Intentions changing	‘I think it was deliberate in terms of there was a bit of concern around risk, and the way I work is pretty rare where I get them to come in and say “let's go” and go through session, session, session. Maybe they have been waiting for the right cases and when you are less confident there is an avoidance and you are more likely to go “oh I'm quite busy”, or “I can't do it because I have this to do”, erm, it's something that I've done. I don't whether there was a delay in referrals, getting cases through.’ (Clinician 103)
		Intentions not to use new intervention	
Goals	Facilitator	Action planning	‘I am leaving this service and she is going away for 3 weeks so I have pulled these 3 sessions together and brought Mum in as co-therapist because even if I was staying she would have had a 3-week gap. She probably would have forgotten a lot of it so hopefully if Mum comes she can remind her and where there is a good relationship with their parent it can really help.’ (Clinician 106)
		After treatment	
Memory, attention and decision-making	Barrier	Training was woolly	‘I think because we are all doing different roles erm, I think it isn't always at the front of people's minds and day-to-day working conditions get in the way. It took, it was a bit slow to start with, to get the forms, the books, the forms, the forms, and I guess you forget if you aren't practicing something you forget.’ (Clinician 108) ‘Other management kind of forget we are doing it. They forget yeah because sometimes I will go in the assessment meeting, and it won't be necessary, so they have kind of forgot that it is running. Yes, so that is a barrier I would say. It's because they are busy.’ (Clinician 107)
		Busy professionals can forget to use it	
		Lack of awareness	
		Decided to use established therapies	
		Case-load and responsibilities diverted attention away from new intervention	
Environmental context and resources	Barrier	Working in the community	‘I think because we are all doing different roles, erm, I think it isn't always at the front of people's minds and day-to-day working conditions get in the way. It took, it was a bit slow to start with, to get the forms, the books, the forms, the forms, and I guess you forget if you aren't practicing something you forget.’ (Clinician 108) ‘I've done session one with him but then he has been off, he's broken his arm and then he was excluded from school and then he was late so missed his session and I found that a little bit and because of the school holidays I've not had a clear run with him, erm, so obviously there are little barriers like that.’ (Clinician 104)
		Working in a clinic	
		Busy	
		Time pressure	
		Limited capacity	
		Changes in job role	
		Lack of appropriate staff	
		Communication	
		Parents	
		Children and young persons’ home environment	
Social influences	Facilitator	Learning from others	‘I think it was useful to come together and speak to people that had already started using it and what challenges they had faced. So, it was really nice and I think we need more of them. Just because if I think it's nice and everyone will deliver it differently and if you have something you are getting stuck on another clinician may have a top tip that has worked really well, I think it's valuable having that space to have a chat.’ (Clinician 106) ‘I think everyone has been quite positive towards it because everyone, I know there are other members that have used manualised interventions and behavioural activation before, they were all quite positive and I think that made me more positive in that I trust the experience of those people.’ (Clinician 104)
		Experienced or unexperienced clinicians	
		Do not feel like the only one	
		Communication between staff is helpful	
		Champions in service	
		What clients expect from therapy	
		Positivity of intervention spreading from others	
Emotion	Facilitator	Excited	‘I was a bit nervous because I have never done any behavioural activation before, erm so I was a bit nervous about it.’ (Clinician 101) ‘Yeah, so, before it I was a little but “ooo” see how it goes, then we got in there and then it went quite smoothly because it goes through step by step, quite easy, so you go through each of these sections.’ (Clinician 102)
		Nervous	
		Anxious	
		Relaxed	
		Comfortable	

TDF, Theoretical Domains Framework; IAPT, Improving Access to Psychological Therapies; CBT, cognitive–behavioural therapy.

#### Beliefs about capabilities and professional role/identity

The beliefs about capabilities domain is defined as ‘the acceptance of the truth or reality of an individual's capabilities or talents which can be put to beneficial use’.^13^ The professional identity and role domain is described as a person's consistent set of behaviours and displayed personal qualities in a work setting. There was a consistent pattern within the data-set of beliefs about capabilities and professional role/identity themes interrelating and acting as either facilitators or barriers to the behaviours involved in implementing the new intervention. For example, MHPs who perceived themselves as new clinicians initially had low confidence in their capabilities of enacting behavioural activation as specified in the manual. However, they perceived practicing using the manual as a way of increasing their capability; therefore, perceiving oneself as a new clinician ultimately facilitated the use of the new intervention.

‘I am a relatively new clinician, I feel at the moment I'm pretty low in confidence, erm, [ … ] I think the next patient I have, as I have more practice, I'll know how to make things a little different or words things differently so it sounds better or erm, just kind of comes across better and maybe I will understand it more. [ … ].’ (Clinician 101, site A)

On the other hand, MHPs who perceived themselves as specialised or experienced clinicians appeared less open to behavioural activation as a new and unfamiliar way of working, feeling more capable with their usual methods. One participant suggested that the introduction of a new intervention may call into question their current methods of practice, and acted as a barrier.

‘[ … ] so when you are less experienced you will go in and just hoover up every skill [ … ] but for specialists like me you are like “I use this, I know this works for me” [ … ] “what if I'm not particularly good at this?” I think it is harder to [ … ] accept it.’ (Clinician 103, site A)

#### Social influences

The TDF describes social influences as the interpersonal processes that can change the way a person thinks, feels and behaves. Social influences were found to be a facilitator in implementing the new behavioural activation intervention; for example, MHPs felt supported within group supervision and training. Coming together as a group made MHPs feel calm about communicating their concerns, and helped individuals overcome challenges when using the intervention, by learning from others.

‘I quite liked group supervision [ … ] you kind of share experiences, and like we are new clinicians and [supervisors] are more experienced [ … ] so it is nice to share our experiences. [ … ] sometimes in 1–1 supervision you feel like as a new clinician especially you can't sort of say everything [ … ].’ (Clinician 102, site A)

‘I think it was useful to come together and speak to people that had already started using it and what challenges they had faced. [ … ] another clinician may have a top tip that has worked really well.’ (Clinician 106, site B)

Additionally, MHPs noted that there were differences in perceived social support within sites and this may have influenced the uptake of the new intervention.

‘[ … ] there are different personalities. I know site B have a different spread of practitioners who are using this compared to ours, they seem to have gone more with [ … ] people with more experience rather than we have given it to assistant psychologists who naturally I think, because their interest is getting on to a clinical psychology doctorate, are quite happy to do a clinical intervention [ … ] and they are quite open and have that mind set. Maybe, for other professionals with different backgrounds it's not a priority.’ (Clinician 103, site A)

One aspect of this could have been the main therapeutic traditions used in the organisations, with site A tending to be used to more integrative therapeutic approaches, so perhaps MHPs felt adopting new ways of working would be supported by peers.^32^

#### Beliefs about consequences

The beliefs about consequences domain refers to how an individual perceives the possible results of performing a certain behaviour, including the characteristics of the outcome and anticipated regret and consequences.^13^ In this study, MHPs perceived the beliefs about consequences domain acted as both a barrier and facilitator to implementing the new behavioural activation intervention. Some MHPs perceived behavioural activation as ‘not being enough’ as a single intervention, thinking that the young people they were working with would require further input after any behavioural activation intervention. They suggested that in some cases, they decided to refer patients directly to a specialised MHP for an alternative treatment, such as CBT or cognitive analytic therapy, rather than a less experienced MHP who would implement the behavioural activation intervention. Therefore, some beliefs about consequences acted as barriers to implementation of the novel behavioural activation manual.

‘Yes, so that is a barrier I would say. I think because even though we say, “yes, we accept comorbidity”, there is always a feeling that it may need more or it needs a case manager that can manage it and do the work, [ … ] And if the thought is that, yes if they see an assistant (psychologist) for 8 weeks but then it's going to need more work, then they might as well pass it on to that other specialised person straight away.’ (Clinician 107, site A)

Also, some MHPs perceived the seemingly simplistic nature of behavioural activation theory and practice could potentially damage their engagement with the young person they were working with. However, several MHPs did perceive the simplicity and practicality of behavioural activation as a strength for engaging with children and young people who may struggle to access or work with their thoughts and feelings.

‘I think whilst the theory of behavioural activation is so simple, [ … ] until you actually start doing it you won't believe it. I remember when I first got introduced to it, I thought “it is going to be hard to explain this to a child without it being patronising.”’ (Clinician 106, site B)

‘[ … ] whereas the simplicity may be a weakness [ … ] it is also a strength. Sometimes kids struggle to understand what they are thinking and feeling and with this, it's really practical [ … ].’ (Clinician 104, site A)

Further, although a perception from a minority of participants, the supervisors of the implementation staff had positive beliefs about the consequences of introducing the new behavioural activation intervention. The clinical psychologist (a supervisor) interviewed perceived benefits of using the new intervention as it had reduced the CBT waiting list, giving more children and young people access to therapy, and felt comfortable giving consent for assistant psychologists (with limited experience) to conduct therapy sessions using the new behavioural activation intervention. Therefore, this perception from the managerial staff was a strong facilitator in implementing the new behavioural activation programme.

‘[ .. ]clients have come off the CBT waiting list, which is brilliant as I manage the [ … ] list and I usually will be looking at giving those young people CBT wait-list. So, it gives me more confidence, to be able to give our clients an intervention by an assistant psychologist who I know is following a manual, receiving supervision, guided through the session, and has limited training.’ (Clinician 109, site B)

#### Memory, attention and decision-making, and environmental context and resources

The memory, attention and decision-making domain is described as the ability to remember information, focus on features of the environment and select between two or more options. The environmental context and resources domain included any characteristics of an individual's situation or environment that may enable or restrict them carrying out a particular behaviour.^13^ In this study, MHPs perceived characteristics of their work environment, such as shortage of time, high workload and working in the community, as a barrier to implementing the new behavioural activation intervention. This barrier identified under the environmental context and resources domain had a negative effect on the memory, attention and decision-making domain as, because of the busy and time restricted nature of the MHPs’ work environment, they forgot about opportunities to implement the new intervention. This was particularly true for MHPs working at site B, as clinician 108 explained.

‘[ … ] I think it isn't always at the front of people's minds and day to day working conditions get in the way. [ … ], and I guess you forget if you aren't practicing something. I'm out in the community [ … ] and I have to be prepared [ … ] and when I first started with the young person, I didn't have everything because they weren't in the pack [ … ]. It would be great if we could kind have them online. [ … ].’ (Clinician 108, site B)

#### Intentions, beliefs about capabilities, and environmental resources and context

The intentions domain is explained as the act of an individual making a conscious decision to perform a behaviour.^13^ Some participants perceived that, although environmental challenges are a real and ever-challenging barrier for those at the front line of care deciding to change their practice, discussion of these may at times overshadow another important but less socially acceptable barrier, such as lack of confidence, or even the feeling that the intervention should not take priority (linking to the beliefs about its value described above). This was insightfully described by clinician 103, as follows:

‘[ … ] I think the delay in using the manual was deliberate. [ .. ] maybe, when you are less confident there is an avoidance and you are more likely to excuse yourself by saying “oh I'm too busy”, or “I can't do it because I have this to do,” erm, it's something that I've done.’ (Clinician 103, site A)

#### Knowledge

Although a perception from a minority of participants, a further barrier faced by a new clinician when using the manual was their lack of knowledge of how to deal with risk in the session. Risk is defined as ‘people who by the nature of their condition, symptoms, experiences, behaviour or lifestyle may be at risk of suicide or self-harm; violence or aggression (including homicide); abuse or neglect (including self-neglect)’.^33^ Although this barrier was perceived by just one participant, it is an important insight that should be highlighted. This barrier was perceived to have the potential to affect other new MHPs because in some services and job roles it was not mandatory to complete risk training, and therefore was perceived as necessary to add to existing training for the new behavioural activation intervention.

‘[ … ] I got to risk, and I haven't been trained on risk yet. Erm, and it's not in my mandatory training and risk came up in the sessions erm, [ … ] I felt quite like something should have been done around risk in the training [ … ]. That felt a little uneasy.’ (Clinician 102, site A)

### Exploring theory-linked techniques to overcome identified implementation barriers

Following the mapping process outlined in the methods section, 39 TDF-linked BCTs were initially identified from the TTT (see Table 2). These selected BCTs were evaluated against the APEASE criteria (see Table 3), to assess the feasibility of these behaviour change interventions within CAMHS. In detail, each BCT was appraised in terms of its affordability, practicality, effectiveness, acceptability, safety and equity, through discussion with the research team, using their academic and contextual service knowledge. For instance, the research team appraised that offering practitioners financial rewards (one of the 93 BCTs of the BCT taxonomy) could be potentially unaffordable and inequitable. Fourteen BCTs were then selected as both theory-linked and feasible in helping overcome MHP-identified barriers to implementing behavioural activation with young people. Practical examples for each BCT are listed in Table 2.

**Table 2:** Mapping Theoretical Domains Framework domains, barriers and facilitators onto behaviour change techniques, to help clinicians implement behavioural activation in routine practice

TDF domain	Reported clinician barrier	Reported clinician facilitator	Theory-linked BCTs mapped and evaluated as feasible	Examples of BCTs in context
Professional identity/role	Identity as a specialised clinician, do not feel need for a behavioural activation manual	Identity as new clinician, more open to using a behavioural activation manual	Social support (unspecified), framing/reframing credible resource	Persuasive positive messages by a trusted source (e.g. study leads or supervisor) to convince staff that this is indeed part of their identity as forward-thinking, flexible, formulation-driven clinicians
Beliefs about capabilities	Specialised clinicians worried that they would not be as good at using the new intervention compared with previously learnt methods	Some clinicians trained in manualised therapy and felt at ease using the new therapy. New clinicians felt weak beliefs about capabilities, but wanted to build on this and practice using the manual	Problem-solving, instruction on how to perform behaviour, demonstration of behaviour, behavioural practice and rehearsal, verbal persuasion about capability, self-talk	The team discussing barriers together and developing solutions that would then be discussed step by step, demonstrated in practice and rehearsed with colleagues, supervisors or study leads to increase clinician beliefs in their capabilities. Supervision would be a useful opportunity to assure clinicians of their capability, reflect on past successes and prompt positive self-talk
Beliefs about consequences	Worried about using it with children with comorbid problems, worried about children and young people finding the simple nature of behavioural activation patronising	Simplicity of behavioural activation can help children who struggle to access or talk about thoughts and emotions, decreases the CBT waiting list	Comparative imagining of future consequences, pros and cons	Clinicians could be prompted to think about reasons for and against using behavioural activation with different young people, including alternatives (e.g. the young person remaining on the waiting list for another therapy). Explore with clinicians the different consequences that may be expected, with the important idea that behavioural activation is ‘simple, but not easy’
Social influences		Group supervision and group training are a source of social support and learning from others.	Social support (unspecified and practical), social comparison	Group training or supervision sessions, with opportunity to access general social support; practical support to help implementation, social comparisons and learning from others about what works and how
Intentions	Avoiding using the intervention		Information about health consequences	Providing useful information about the potential positive consequences of the intervention; many other BCTs would help to ultimately influence clinician intentions
Memory, attention and decision-making	Professionals forget/are not aware, decide to use established therapies, case-load and responsibilities occupy attention		Prompts and cues, adding objects to the environment	Prompts and cues such as email reminders, post-it notes or regular prompts in meetings to ‘think behavioural activation’ when formulating and treatment planning in the community
Environmental context and resources	Working in the community; busy, time pressure; limited capacity; changes in job role		Social support (practical), adding objects to the environment, prompts and cues	Supervisors to add a box of printed sessions of the behavioural activation manual, activity scheduling sheets, information sheets for parents, extra worksheets in the workplace; providing online versions
Skills	No previous therapeutic experience, feeling like you have to cover everything in the manual	Previous experience of using a manual, using the manual flexibly, gaining new skills	Behavioural practice/ rehearsal	As above, offering training, including instructions and practice/rehearsal to increase skill and confidence with different levels of detail
Knowledge	No training around risk		Instruction on how to perform behaviour, behavioural practice and rehearsal	Practical, behaviourally informed education initiatives to develop knowledge of risk including different forms and types, risk factors, and practicing ways of handling risk in a therapeutic session

TDF, Theoretical Domains Framework; BCT, behaviour change technique; CBT, cognitive–behavioural therapy.

**Table 3:** Identifying barriers and facilitators for a new manualised behavioural activation intervention using the Theoretical Domains Framework, mapping these to behavioural change techniques and appraising the techniques with the APEASE criteria

Target behaviour	TDF domain	Reported barrier	Reported facilitator	BCT	APEASE criteria check
Implementation of the new manualised behavioural activation intervention	Professional identity/role	People who viewed themselves as specialised clinicians.	People who viewed themselves as new clinicians	Social support (non-specific)	Yes
				Social comparison	No
				Social pressure	Yes
				Credible resource	Yes
	Beliefs about capabilities	Specialised clinicians worried that they would not be as good at using the new intervention compared with previously learnt methods	Some clinicians trained in manualised therapy and felt at ease using the new one. New clinicians felt weak beliefs about capabilities but wanted to build on this and practice using the manual	Goal-setting	No
				Problem-solving	Yes
				Biofeedback	No
				Instruction on how to perform	Yes
				Demonstration of behaviour	Yes
				Behavioural practice and rehearsal	Yes
				Graded tasks	No
				Verbal persuasion about capabilities	No
				Focus on past success	No
				Self-talk	Yes
	Beliefs about consequences	Worried about using it with children with comorbid problems, worried about children and young people finding the simple nature of behavioural activation patronising	Simplicity of behavioural activation can help children who struggle to access or talk about thoughts and emotions, decreases the CBT waiting list	Information about health consequences	No
				Salience of consequences	No
				Information and social and environmental consequences	No
				Anticipated regret	No
				Pros and cons	Yes
				Comparative imagining of future consequences	Yes
				Comparative imagining of future consequences	No
				Reward	No
	Social influences		Group supervision and group training a source of social support and learning from others.	Social support (unspecified)	Yes
				Social support (practical)	Yes
				Social comparison	Yes
				Social reward	No
	Intentions	Avoiding using the intervention		Goal-setting	No
				Information about health consequences	No
				Incentive	No
	Memory, attention and decision-making	Professionals forget to use it or are not aware of it, decide to use established therapies, case-load and responsibilities occupy attention		Conserving mental resources	No
				Prompts and cues	Yes
	Environmental context and resources	Working in the community, busy, time pressure, limited capacity, changes in job role		Social support (practical)	Yes
				Prompts and cues	Yes
				Remove adverse stimuli	No
				Restructuring environment	No
				Avoidance/reducing exposure to cues for behaviour	No
				Adding objects to the environment	Yes
	Skills	No previous therapeutic experience, feeling like you have to cover everything in the manual	Previous experience of using a manual, using the manual flexibly, gaining new skills	Graded tasks	No
				Behavioural practice/rehearsal	Yes
				Instruction on how to perform	Yes
	Knowledge	No training around risk		Instruction on how to perform behaviour	Yes
				Behavioural practice and rehearsal	Yes
				Information about antecedents	No
				Biofeedback	No
				Information about environmental and social consequences	No
				Information about health consequences	No

TDF, Theoretical Domains Framework; BCT, behaviour change technique; APEASE, affordability, practicality, effectiveness, acceptability, side-effects, equity; CBT, cognitive–behavioural therapy.

## Discussion

This study explored influences on MHPs’ self-reported practice behaviour when implementing behavioural activation interventions with young people in routine care. As a therapy with growing evidence for young people, which may be recommended in future clinical guidelines, it is vital to consider barriers and facilitators to its feasible implementation routine practice. Barriers and facilitators mapped onto 13 of the 14 domains of the TDF, a behavioural science framework used to explore theoretical influences on behaviour.^14^ Using the new TTT tool^18^ and APPEASE criteria,^30^ these were linked to 14 evidence-based BCTs that could be integrated within routine training, meetings and supervision to help service leaders to implement behavioural activation successfully within a CAMHS.

MHP accounts suggested that their perceptions of their professional identity/role and their beliefs about capabilities to use behavioural activation were important influences on their practice. Generally, this links to other implementation science studies exploring new clinical pathways in hospitals, which have found that MHPs can be hesitant where there is a perceived mismatch with their professional identity/role^34^ and capabilities.^35^ More specifically, our study found that participants viewed behavioural activation as a therapy that would be most appropriate for use by less experienced and more junior therapeutic practitioners. This was both because of the perceived ‘simple’ theoretical nature of behavioural activation itself and the detailed manuals provided by study leaders. This links closely with the global public health agenda and the need to build capacity through engaging a wider psychological workforce in delivering mental healthcare.^36^

Additionally, in this study, MHPs’ beliefs about the consequences of using behavioural activation (how successful it would be and unintended effects) operated as both a facilitator and barrier to implementation. This is important as health professionals’ personal attitudes to new treatments and therapies have been found to play a role in how widely they are adopted in other studies of their perceptions.^37^ Specifically, some MHPs viewed behavioural activation as ‘too simplistic’ to work with their young people with depression, especially clients with other comorbid mental health problems. One previous study of adult patient perceptions of behavioural activation identified that some perceived the therapy as ‘simplistic’ and this could be a treatment engagement barrier.^38^ However, many participants in our study were somewhat surprised to find that their young clients engaged well with the approach, and more fully understood its theory compared with more ‘complex’ therapies, like CBT.

MHPs also perceived environmental context and resources as an important influence on their habitual use of behavioural activation with clients (e.g. forgetting or having little time to take opportunities to use the new behavioural activation approach). Psychological theory and research asserts that many clinical behaviours are controlled by automatic process, and are determined by habit and context rather than clinicians’ deliberative thinking.^39^ Action and coping planning techniques are recommended to help practitioners ‘plan to be routine’, and are among the implementation strategies recommended In this study.^40^ However, some MHPs in the study questioned whether lack of time was a ‘real barrier’ for MHPs in delivering behavioural activation, suggesting other underlying barriers could be more relevant. Other studies exploring healthcare practitioner practices have also suggested that an expressed lack of time for an activity can sometimes be a proxy for other barriers, such as lack of confidence or a perception that the activity has little value.^41^

Above all, perceptions of barriers and facilitators were idiosyncratic, and there were several interacting themes and domains. This indicates the complex nature of implementing new therapies and services in routine practice and the importance of exploring MHP accounts. Exploring identified barriers and facilitators and linking these to theory-linked BCTs has been shown to increase the impact of healthcare professional training and education in physical health services in the UK and in low- and middle-income countries (e.g. Bull et al^42^).

### Strengths and limitations

This study purposively sampled a range of MHPs to gather an in-depth and wide range of perspectives and experiences. It was part of a wider feasibility study.^20^ This study adds to the limited research that has used the TDF to investigate barriers and facilitators to implementing psychological therapies,^9,43^ and, to our knowledge, is the first study to specifically investigate the implementation of a behavioural activation intervention. A strength of this study is that it demonstrated how the novel TTT can be used to systematically identify and link TDF domains to evidence-based BCTs, which can be used to overcome barriers and support facilitators of implementing a new intervention into clinical practice. BCTs were appraised for suitability in the specific service by using the APEASE criteria to help bridge the gap between BCTs that are evidence-based and clinicians’ accounts of what may actually be feasible implementation strategies.^28^ Using tools like the TTT and APEASE criteria has been suggested to support the use of the TDF in the future, and overcome the common barriers to using the TDF experienced by researchers, such as linking identified TDF domains to BCTs and developing implementation interventions to support uptake of evidence-based practices.^44^ A limitation to using the TDF was the amount of time the framework requires from researchers, a constraint echoed in other implementation studies using the TDF.^44^ Also, there is no guidance about how to deal with complexities in a data-set; for example, a number of barriers perceived by staff involved multiple domains, whereas most BCTs target domains individually.^37^ Finally, the small sample size of NHS staff studied and their variation in experience of using behavioural activation limits the generalisability of the findings.

In conclusion, this qualitative study applied the TDF and other novel behavioural science methods to understand perceived barriers and facilitators to implementing behavioural activation for young people, and mapped these to evidence-based and feasible BCTs. These could be applied in future implementation of behavioural activation interventions to overcome the barriers and facilitators and support uptake within widespread clinical practice. Ultimately, this may help to improve access to psychological therapies for children and young people with depression, and enhance the uptake of research into practice.

## Data Availability

The data that support the findings of this study are available from the corresponding author, K.W., upon reasonable request.

## Supplementary material

For supplementary material accompanying this paper visit http://doi.org/10.1192/bjo.2022.7. click here to view supplementary material
