# Supplementary material for: Exploring barriers and facilitators for mental health professionals delivering behavioural activation to young people with depression: qualitative study using the Theoretical Domains Framework
Source: BJPsych Open. 2022 Feb 4;8(2):e38. doi: 10.1192/bjo.2022.7 (PMC8867899; doi:10.1192/bjo.2022.7)
Supplement: Supplementary file 1 [file bjosup.zip › S2056472422000072sup001.docx]

1. **How did you feel before implementing the BA intervention?**

*Probes:*

- 1. *What were your thoughts?*
  2. *It’s potential?*
  3. *Confidence of implementation?*
  4. *Overall staff feeling?*

1. **Please could you tell me about your experiences of implementing the BA intervention with children and young people with low mood?**

*Probes*

- 1. *What did you think of the BA intervention?*
  2. *How acceptable? Tell me more.*
  3. *Are these characteristics different compared to other treatments given to CYP with low mood?*
  4. *What did it feel like giving the BA intervention to CYP?*

1. **The BA intervention manual contains certain therapeutic strategies and techniques. Could you tell me which elements of the therapy you used most?**

*Probes*

- 1. *Why?*
  2. *Are there any elements you frequently used?*
  3. *Were there elements you did not like?*

1. **Could you tell me if and how the intervention may have changed aspects of your work?**

*Probes:*

- 1. *Have you taken anything away from the BA intervention?*
  2. *Has your working style adapted/ changed?*
  3. *Changed your way of thinking about treatment for depression in CYP?*
  4. *Was the BA intervention noticeably different to the treatments currently used?*

1. **Were there times when the BA intervention was difficult to use?**

*Probes:*

- 1. *Personal contextual factors*
  2. *Environmental factors*
  3. *Therapeutic relationship factors*
  4. *Stages or exercises causing difficulty*
  5. *Any that could have been done to overcome these difficulties?*

1. **Could you tell me whether you perceived any positives of using the BA manual?**

*Probes:*

- 1. *Perceived outcomes for patients*
  2. *Manual*
  3. *Stages and exercises*
  4. *Homework tasks*
  5. *Process*
  6. *Confidence of using the manual*
  7. *Motivation – does BA work?*

1. **Could you tell me how others found the BA intervention?**

*Probes:*

- 1. *Patients*
  2. *Staff*
  3. *Supervisors and management*

1. **Do you think there was satisfactory training and guidance in using the BA manual?**

*Probes:*

- 1. *Is this your first-time using BA?*
  2. *Enough help?*
  3. *Enough resources?*
  4. *Enough explanation and clear guidance within the manual itself?*

1. **Have you had any supervision whilst using the BA intervention?**

*Probes:*

- 1. *Group/ individual?*
  2. *How often?*
  3. *Was it helpful?*
  4. *If group supervision, was there any problems practitioners were facing unanimously?*

1. **Finally, is there anything else you would like to talk about on your experiences of using the BA intervention?**

*Probes:*

- 1. *Any memorable parts of using BA?*
  2. *Any specific characteristics that made the intervention effective or hard to use?*
  3. *Do you have any suggestions for future trial?*
